# Modeling the Effects of Vaccination and Treatment With Third-Generation Macrolides or Oxytetracyclines on Persistence and Impact of Contagious Bovine Pleuropneumonia

**DOI:** 10.1155/tbed/1831782

**Published:** 2025-06-04

**Authors:** Jeffrey C. Mariner, Hezron Wesonga, Geoffrey Muuka, Kristin Stuke, Angela Colston, Stephen Wilson

**Affiliations:** ^1^Buak Chan Epi and Livestock, Chiang Mai, Thailand; ^2^Veterinary Science Research Institute (VSRI), Kikuyu 00902, Kenya; ^3^Central Veterinary Research Institute (CVRI) Ministry of Fisheries and Livestock, Lusaka, Zambia; ^4^Global Alliance for Livestock Veterinary Medicines, Nairobi, Kenya; ^5^Global Alliance for Livestock Veterinary Medicines, Edinburgh, UK

## Abstract

This research assesses the impact of contagious bovine pleuropneumonia (CBPP) vaccination, treatment, and combined vaccination and treatment at the herd and population level in heterogeneous, stochastic, state-transition models of CBPP transmission. Results from published trials with oxytetracycline or third-generation macrolides (tulathromycin and gamithromycin) were used to inform parameters for antibiotic treatment. Societies have evolved and the levels of movement control responsible for the previous success of vaccination programs are no longer possible. Current vaccines, when applied in the absence of movement control, did not result in eradication. For hypothetical vaccines with 85%–95% efficacy and 3 years duration of immunity, more than 3 years of biannual vaccination would be required to reduce herd prevalence to near eradication levels. The results of treatment scenarios indicated that small-scale, focused community-based programs working through trained community members to systematically detect and treat suspect cases with oxytetracyclines or third-generation macrolides can eliminate CBPP from defined endemic populations within a period of 6 months. Oxytetracylcines are effective, inexpensive, and widely available. The rapid clinical response to the third-generation macrolides is an additional, direct incentive of interest to livestock owners and has the potential to entirely change the economics of CBPP control programs. Development and validation of effective, practical treatment protocols have the potential to reduce total antibiotic use over the current situation of widespread, haphazard use of antibiotics and enhance antibiotic stewardship. Combined programs covering regions that promote treatment of clinical cases and vaccination of the contact population at risk are of interest. Large scale treatment and vaccination approaches have the potential to eliminate infection in time frames of 2–3 years. In the future, pilot control programs based on public–private-community partnerships should be implemented at the community level that addresses the technical strategy, the modern institutional and socioeconomic challenges, and new opportunities for control.

## 1. Introduction

Standard control methods that have been successfully used to eradicate contagious bovine pleuropneumonia (CBPP) in many parts of the world rely on isolation and culling of infected animals. However, insufficient veterinary surveillance and limited budgets for compensation mean that this is not a viable option in most developing countries. The current vaccines available for CBPP, such as T1/44, have limited efficacy and duration of immunity [1], and vaccination in the absence of other control measures, such as stringent movement control, has had limited impact.

Important advances were made in the control of CBPP due to *Mycoplasma mycoides* subspecies *mycoides* (small colony) in the 20th century. The disease was suppressed or even eliminated throughout much of its range. However, in recent years, conventional CBPP control approaches have broken down for reasons that are primarily related to changing societal and animal health institutional realities that preclude the implementation of strict movement control and effective publically-funded mass vaccination programs. Strategies that once worked are no longer socioeconomically feasible to implement and CBPP is now one of the principal constraints to pastoral livelihoods and an intractable institutional problem for many countries.

Over 60 years of research has sought to enhance tools available for CBPP control. Despite considerable investment and the evaluation of several candidate vaccines, improved CBPP vaccines have not been developed, and basic science challenges remain in the development of more effective vaccines [1]. Public investment in CBPP control is limited and, for the most part, has failed to sustain control with vaccines alone. Although officially discouraged, treatment with antibiotics is a common intervention used in the control of CBPP in the field today. This is due in part to limited access to vaccines resulting from public control of vaccine supplies while antibiotics are available in the marketplace. Given the progress in democratic systems of governance and greater political empowerment of rural communities, movement control, which was an integral part of the success of control programs, is largely no longer feasible at the level needed to be effective for an issue such as CBPP. The failure of CBPP control is primarily a failure of policy to evolve with socioeconomic context. More recent research has focused on the development of rational intervention strategies that capture private incentives and establish an evidence base for responsible and effective use of antibiotics and vaccines.

Global Alliance for Livestock Veterinary Medicines (GALVmed) research has made available important new data on vaccines and treatments for CBPP [2]. The results reaffirm that the T1/44 vaccine remains the preferred vaccine available after 60 years. T1/44 vaccine (Peribov, BVI, Botswana) was found to reduce significant lesions by 87% in animals challenged 6 months postvaccination and was associated with only minor vaccinal reactions. Novel third-generation macrolide antimicrobials have been adopted widely in European Union and United States of America during the last 10 years for treatment of bacterial and mycoplasma (non-CBPP) respiratory diseases in cattle. The GALVmed research found that oxytetracycline and these third-generation macrolides were highly efficacious antibiotics in the treatment of clinical CBPP [3]. Most importantly, in a controlled study, the third-generation macrolides and oxytetracycline reduced transmission from treated animals to levels consistent with the elimination of CBPP infection from the population under treatment. The results are fully consistent with earlier studies on the T1/44 vaccine and the impact of treatment on transmission [3, 4]. The decisive clinical response is a new development associated with the third-generation macrolides that are of direct benefit to livestock owners and can serve as an important driver of livestock-owner investment in control programs.

The present modeling study integrates data from the published GALVmed-sponsored studies on the efficacy of treatment and vaccination for the control of CBPP into existing models of CBPP transmission and control intervention impact [3, 4]. These models were built on detailed reviews of the literature on the epidemiology of CBPP and parametrized with original field observations of livestock owners on the behavior of CBPP in South Sudan and Tanzania. The original studies focused on assessing the impact of vaccination and treatment.

The objective of the modeling conducted for GALVmed was to determine the implications of the newly published antibiotic research findings for practical control options for CBPP. The modeling looked at different field scenarios in endemic settings and predicted the impact of both existing control practices and potential innovations in control practices based on the evidence-based estimates of vaccine and treatment efficacy produced by the GALVmed research. The analysis examines a comprehensive range of vaccination and treatment options, including combined programs of treatment of clinical cases and vaccination of the remainder of the contact population at risk.

From the perspective of disease control and eradication, the important parameter of interest is the effect of vaccines and treatment on the transmission of infection, resulting in new *infectious* individuals. More specifically, the value of the intervention is assessed by its ability to prevent the development of new individuals capable of onward transmission of exposure. It is important to note that individuals exposed to or who contract a transient infection that does not lead to shedding of the agent are not *infectious*, and such dead-end infections are not of epidemiological interest in disease control. The effects of treatment, vaccination, or combinations of interventions were assessed by comparing the following metrics:

• Determination of the effective reproductive number (*R*_*e*_) for the treatment scenarios.
• Time to elimination from a set herd size for each intervention scenario.
• Final herd prevalence of infection and total mortality due to CBPP experienced by the population over the duration of the simulation.

The paper concludes with suggestions for piloting of promising intervention strategies at the community scale in endemic settings.

## 2. Methodology

### 2.1. The Susceptible, Vaccinated, Exposed, Infectious, Sequestra, Recovered (SVEIQR) Model for CBPP Transmission

**Figure 1:**
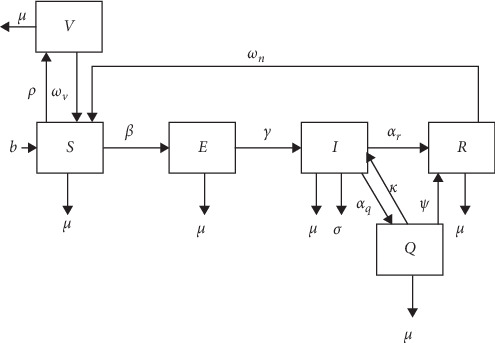
Diagram of the CBPP SVEIQR model structure illustrating the relationship between the six model states and the parameters that described transition pathways [3]. Nonspecific mortality (*μ*) occurred in all six states. Births (*b*) entered the susceptible (*S*) state. The rate at which animals moved from the susceptible to the exposed state (*E*) was governed by the effective contact rate (*β*). Exposed animals are infected but not yet infected. Only infectious (*I*) animals experienced CBPP mortality (*σ*). The rate at which exposed animals became infectious was described by *γ*. Immunization was modeled as a transition from the susceptible state directly to the vaccinal immune state (*V*) at the immunization rate (*ρ*). Vaccinal immunity was lost at the rate *ω_v_*. Infectious animals could recover fully and directly enter the recovered state (*R*) at the rate *α*_*r*_ or develop infected sequestra (*Q*) at the rate *α*_*q*_. The sequestra of animals in the *Q* state either became sterile, and the individual entered the *R* state at the rate *ψ* or was reactivated at the rate *κ*, and the individual re-entered the infectious state. Animals in the *R* state were naturally immune but may possess sterile sequestra. Naturally, immune animals could lose their immunity at the rate *ω*_*n*_ and return to the susceptible state.

The model design and structure are fully described previously [3, 4], and this section summarizes key aspects for the reader's convenience. The SVEIQR CBPP model is a heterogeneous population, state-transition, stochastic model. Note that *S* and *E* stand for susceptible and exposed classes, respectively, and *I* stands for the infectious class. Exposed animals are infected but not yet infectious. The *Q* class is noninfectious sequestra, and the *R* class is recovered animals that are reported to be immune for the reminder of their life. As vaccinal immunity is transient, a separate vaccinated state, *V* is included in the model. The model and derivation of parameter estimates from the literature and field research are well described [3, 4]. The model structure is presented in Figure 1, and the parameter definitions are summarized in Table 1.

Equation 1 below expresses the relationship between *R*_0_, beta, and the model parameters determining the probability that an animal survives the latent period and duration of the infectious period:

(1) $$\begin{array}{c} R_{0}=\beta \frac{\gamma }{\left(\gamma +\;µ\right)\left(\;µ+\alpha_{r}+\alpha_{q}+\sigma \right)}. \end{array}$$

For the development of the original SVEIQR model, the value of the basic reproductive number was estimated from the average age of infection in endemic settings from serological data. These estimates from the field were compared with the value of *R*_0_ calculated from the model parameters. This same equation was used to calculate the effective reproductive number (*R*_*e*_) when the parameters were adjusted to simulate the effect of treatment.

The parameters used in the baseline model gave a distribution of values of *R*_0_, where 90% fell between 3.4 and 4.7, and the mean was 4.1. These values of *R*_0_ agree well with those predicted by Mariner et al. [3] from available serological data [5–7]. Figure 1 presents the model structure: states and parameters governing transitions between states.

Seasonal variations were modeled by a periodic trigonometric function to modulate the values of *β* [8]. This was modeled as follows:

(2) $$\begin{array}{c} \beta \left(t\right)=\beta_{0}\left[1+A\;\sin\left(2\pi t\right)\right], \end{array}$$

where *β*_0_ is the average effective contact rate, and *A* is the amplitude of the seasonal effect. The parameter *A* could be adjusted by changing a single cell value (Table 1). The magnitude of seasonality could be varied by entering a fraction between 0 and 1.

**Figure 2:**
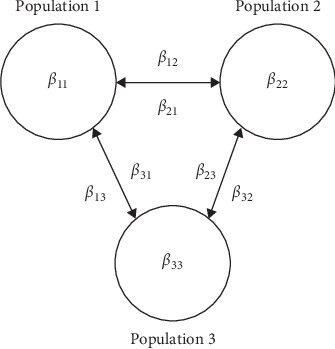
Diagram of the three subpopulations and *β* transmission parameters for within-herd transmission and between-herd transmission. Population 1 was the reference population, and Populations 2 and 3 were in-contact herds. All populations were given a size of 500 heads, and the ratio of within-herd to between-herd *β* transmission parameters was set to 0.1 for all simulations.

The model simulates three interlinked subpopulations, each set to 500 heads to approximate pastoral herds in Africa. There are *β* transmission parameters for within-herd transmission and between-herd transmission. The all within and between herd parameters can be set individually to allow modeling of the impact of herd-specific programs in an untreated population (Figure 2). The effect of different subpopulation sizes and the ratio of within-herd contact rates to between-herd contact rates (*η*) was examined in detail in previous work. Equal population sizes of 500 heads and a ratio of contact rates (*β*) of 0.1 was found to best reproduce field conditions [4].

### 2.2. Vaccination

The GALVmed-sponsored research found 87% reduction in modified Hudson and Turners scores (calculated for the size and type of lung lesions [9]) when T1/44 vaccinates were compared to controls after challenge with virulent CBPP at 6 months postvaccination [10]. A previous review of the literature [11–17] found efficacy levels in the range of 50%–80% and durations of immunity of up to 2 years [3].

For vaccination, the immunization proportion (*ρ*) equals the vaccination efficacy (*p*_*v*_) multiplied by vaccination efficiency (*p*_*e*_) and the vaccination coverage (*ε*). Vaccination coverage and efficiency refer to the proportion of animals vaccinated and the proportion of animals vaccinated that were correctly vaccinated, respectively.

(3) $$\begin{array}{c} \rho =p_{v}p_{e}\varepsilon . \end{array}$$

The loss of immunity due to vaccination (*ω_v_*) is the inverse of the duration of immunity. The method used to model the loss of immunity in the model was modified from a simple exponential decline [3] to a gamma distribution using the method of stages [18, 19]. Eight subclasses for the immune state were added with an identical rate of loss of immunity (*ω_v_*) for each state.

A sensitivity analysis was run by sampling PERT distributions for the duration of immunity with a range of 12–48 months and vaccine efficacy with a range of 60%–90%. Thereafter, simulations were run for a series of annual and biannual mass vaccination programs where all three linked populations were vaccinated at the same proportion. In these mass vaccination scenarios, vaccination coverage and efficiency were both assumed to be 80%. Combinations of durations of immunity of 1 year or 2–3 years and vaccine efficacy of 50%–80%, 60%–80%, 70%–80%, or 85%–95% were explored in vaccination programs lasting 2–5 years.

Elective vaccination was modeled as vaccination of the reference population but not the two contact populations. Vaccination efficiency and coverage were set to 0.9 for elective vaccination.

The proportion of reference herds that remained infected at the end of the 6-year simulation (final herd prevalence), the time until the first fade out of infection from the reference herd, and the mean total mortality due to CBPP in the reference herd are reported.

### 2.3. Treatment

The impact of treatment on transmission was estimated from the evidence of effective transmission to the sentinels placed in contact 30-days posttreatment with the four groups of infected treated animals (saline-treated infected controls [Group 1], tulathromycin [Group 2], gamithromycin [Group 3], and oxytetracycline [Group 4]) in the two separate experiments (Afade and Caprivi strains) conducted by GALVmed (3,4). Each group of sentinels (Groups 5–1 to 5–4) consisted of five animals except for the gamithromycin and tulathromycin groups in the Caprivi challenge experiment which consisted of four animals. A summary of the data from the studies where sentinel animals were placed in contact with the treatment groups is presented in Tables 2 and 3 and analyzed from the perspective of transmission, resulting in *infectious* cases in the results.

The impact of treatment was modeled as reductions in the infectious period. The following formula defines the relation between these terms:

(4) $$\begin{array}{c} \beta =C\times p\text{ and }R_{0}=\beta \times d, \end{array}$$

(5) $$\begin{array}{c} R_{0}=C\times p\times d. \end{array}$$

The effective contact rate (*β*), which governs the rate (per time) at which susceptible hosts transition to the exposed state, is made up of the probability of a physical contact (*C*) between a susceptible host and an infectious host multiplied by the probability that the contact be an infectious and susceptible individual result in infection (*p*). When the effective contact rate is multiplied by the duration of infection (*d*), it gives the number of new infections resulting in one generational cycle result from one infected host (*R*_0_).

Treatment that shortens the clinical course of cases can reduce the infectious load in the host, the duration of the infectious period, and the level of shedding of the infectious agent. In the model, the effect of treatment was modeled by increasing the recovery rate *α*, which is equivalent to decreasing the duration of the infectious period (*d*). In the SVEIQR model, there are two routes to recovery and two recovery rates. An infected state can either move directly into the recovered state *R* without developing sequestra at the rate *α*_*r*_, or they develop sequestra at the rate *α*_*q*_, which then slowly resolves. The effect of treatment was modeled by increasing both *α_r_* and *α_q_* equally. The dynamics of the sequestra state *Q* were not changed.

Using this method, 56, 42, 28, 21, 14, 10.5, and 7 days were evaluated as the most likely values in the PERT distributions used for the value of *d* or 1/*α*.

Treatment was modeled either as a public program of treatment of all clinical cases in all herds or as an elective treatment where individual herd owners engaged in treatment of all clinical cases in their herd. Mass treatment of all animals, regardless of their clinical status, was not modeled. Elective treatment modeled as reductions in the duration of infection in one reference herd without any change to the two in-contact herds. The same values of d or 1/*α* were used in the reference herd as above.

### 2.4. Integrated Programs of Treatment and Vaccination

The effect of integrated control programs that incorporated treatment of clinical cases and vaccination of contact populations at risk was examined by conducting simulations that incorporated vaccination and increased recovery rates (*α*) (equivalent to reduced durations of the infectious period). Sets of vaccination programs were analyzed with the following combinations of efficacy and duration of immunity:

• 85%–95% efficacy with a duration of immunity of 2 years (*ω_v_* = 0.01).
• 85%–95% efficacy with a duration of immunity of 3 years (*ω_v_* = 0.005).
• 60%–90% efficacy with a duration of immunity of 2 years (*ω_v_* = 0.01).
• 60%–90% efficacy with a duration of immunity of 3 years (*ω_v_* = 0.005).

All vaccination programs were evaluated where the duration of the infectious period was set to the default values (most likely value of 56 days), and values reduced to 75% and 50% of the default duration, most likely values of 42 and 28 days, respectively.

The sets of vaccination programs modeled included annual or biannual vaccinations that lasted 2, 3, and 5 years.

Elective programs of treatment and vaccination (sick animals were treated, and healthy members of the herd were vaccinated) were modeled. For elective programs, treatment and vaccination were only applied to the reference population and no interventions were conducted in the two in-contact populations. Values of 56, 42, 28, 21, 14, 10.5, and 7 days were evaluated as the most likely values in the PERT distributions used for the value of d or 1/*α*. The vaccination program consisted of 5 years of annual vaccination using a vaccine with 85%–95% efficacy and 3 years duration of immunity.

## 3. Results

### 3.1. Vaccination

**Figure 3:**
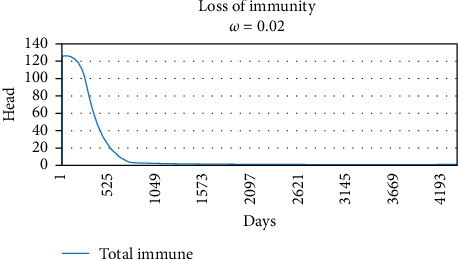
Gamma decay of vaccinal immunity when *ω*_*v*_ set to 0.02. The curve exhibits a plateau of immunity lasting 6 months, followed by a period of rapid decline with 50% loss of immunity 1 year postvaccination.

**Figure 4:**
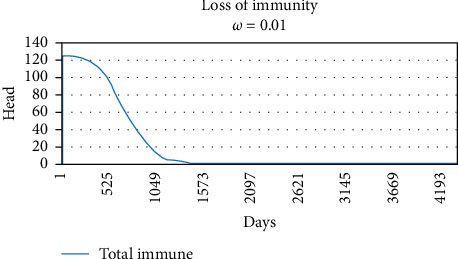
Gamma decay of vaccinal immunity when *ω_v_* set to 0.01. The curve exhibits a plateau of immunity lasting 12 months, followed by a period of rapid decline with 50% loss of immunity 2 years postvaccination.

**Figure 5:**
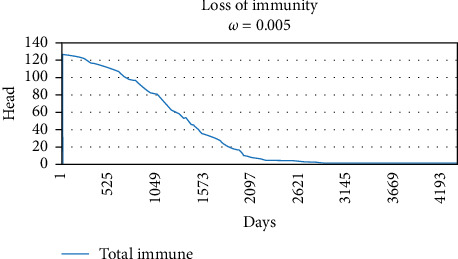
Gamma decay of vaccinal immunity when *ω_v_* set to 0.005. The curve exhibits a slight loss of immunity lasting 2 years, followed by a period of moderate decline with 50% loss of immunity 3 years postvaccination.

Graphs of the loss of immunity following a single pulse of vaccination on Day 1 for three values of *ω_v_* are shown in Figures 3–5, 0.02, 0.01, and 0.005, respectively. The values of *ω_v_* of 0.02, 0.01, and 0.005 correspond to durations of immunity of 1 year, 2 years, and 3 years.

**Figure 6:**
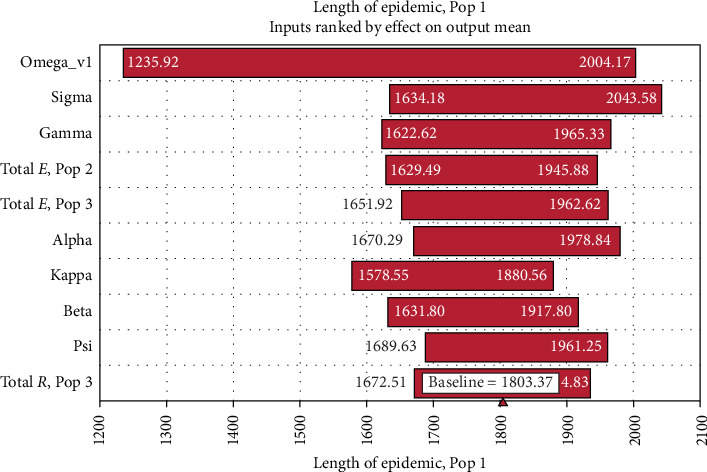
Sensitivity analysis of the effect of parameters and states on the mean length of the outbreak. Only those with the largest effect are shown. The bars indicate the range of length of epidemics output by the model in days in the reference population (Pop 1) resulting from the input distribution of the parameter. Pops 2 and 3 are in the in-contact populations. Total *E* and total *R* are the overall number of exposed and recovered that developed in Pops 2 and 3 during the simulations. The fact that the length of the outbreak in the reference population was sensitive to the number exposed and recovered states in the in-contact populations suggests that a high level of involvement of the in-contact populations contributed to the length of epidemics in the reference population. Alpha (*α*), kappa (*κ*), beta (*β*), and psi (*ψ*) are variables from the model as defined in Figure 1 and Table 1. The length of the outbreak is most sensitive to the value of omega _*v* (*ω_v_*) (the parameter that sets the vaccination duration of immunity). The values of *ω_v_* ranged between 0.005 and 0.02 (1–3 years). Efficacy was set to 60%–90%, with a most likely value of 75%.

Figure 6 presents a sensitivity tornado graph of the parameters and initial conditions with the greatest impact on the duration of infection in an annual vaccination program lasting for 5 years. The duration of the outbreak was most sensitive to the rate of loss of immunity (*ω_v_*) when this parameter ranged across a distribution equivalent to 1–3-year duration of immunity. In a separate tornado analysis of the final herd prevalence, the final herd prevalence was also found to be most dependent on the duration of immunity. Although the efficacy of the vaccine ranged between 60% and 90% in the same simulation, the outcomes were much less sensitive to the value of vaccine efficacy.

The results of simulations where the duration of immunity was set to 1 year (*ω_v_* = 0.02) and 3 years (*ω_v_* = 0.005) are presented in Tables 4 and 5, respectively. Mass vaccination with a vaccine with 1-year duration of immunity was unable to reduce the herd prevalence infection (percent of herds that harbored infection) below 61.8% even when the efficacy of the vaccine was assumed to range between 85% and 95% with a most likely value of 90%, and vaccination was implemented twice per year over 5 years.

When biannual vaccination was carried out over 5 years with a vaccine of 70%–80% efficacy and 3 years (*ω_v_*_ =_ 0.005) duration of immunity, the final herd prevalence was reduced to 2.2%. When vaccine efficacy was increased to 85%–95%, the final herd prevalence was reduced to 0.4% (Table 5).

The results of elective vaccination of the reference herd only are presented in Table 6. Reductions in herd prevalence were moderate, in part due to reinfections after clearance of CBPP from the herd on one or more occasions. On the other hand, the mortality resulting from CBPP was reduced from 179 head to 67 and 41 head for the 5 year annual and biannual vaccination programs, respectively.

**Figure 7:**
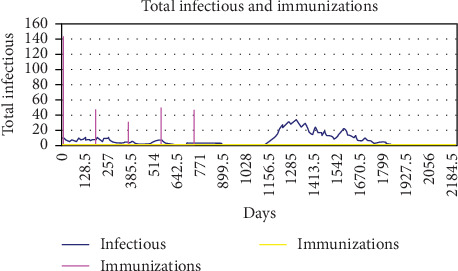
Biannual vaccination in the reference population with a vaccine efficacy between 0.85 and 0.95 and a vaccination coverage and efficiency of 0.9. As this was elective vaccination, it was modeled as pulsed vaccination on single days (purple spikes) as opposed to a continuous mass vaccination approach (yellow line displaying zero vaccinations here). The infection is cleared from the reference population by the beginning of year 3 and reintroduced from the contact populations at the beginning of year 4.

Figure 7 is a sample plot of the number of vaccinations and infectious animals from the reference population of one of the elective vaccination simulations. Note that vaccination clears infection from the herd, but infection is later reintroduced from one of the contact herds.

### 3.2. Treatment

In the GALVmed-sponsored trials, one of the sentinels in each of the trials (20%) put in-contact with infected, untreated controls became positive for the full range of indicators of CBPP infection (postmortem examination, culture, and serology).

Of the sentinel animals in contact with the infected groups treated with third-generation macrolides, one sentinel animal in the group exposed to the group animals infected with the Caprivi strain and treated with tulathromycin showed a complement fixation titer (CFT) of 80 at one time point. Postmortem examination and culture were negative in this animal. In the case of the Afade study, one sentinel exposed to gamithromycin-treated infected animals and one sentinel exposed to tulathromycin-infected animals each developed a CFT of 10. Neither animal was positive on culture nor had evidence of lesions at postmortem. Although the sample size is small and should be treated with caution, it is unlikely that any of the sentinels in contact with the animals that were treated with third-generation macrolides became infectious. The evidence is consistent with the effective reproductive number (*R*_*e*_) for animals treated with tulathromycin and gamithromycin of less than one and approaching zero.

In the case of oxytetracycline, there was evidence of transmission from infected, treated animals to sentinels with both the Afade and Caprivi challenge strains. Two sentinel animals (40%) were ELISA-positive in the Afade experiment but negative on CFT, postmortem, and culture. In the Caprivi experiment, one sentinel animal was positive on nasal swab culture, and another animal was transiently positive by ELISA. This suggests that some animals were exposed but did not become sufficiently infected to sustain transmission. When compared to the amount of transmission from untreated controls, this is consistent with a reduction in transmission to an *R*_*e*_ of less than 1.

The results of increasing the recovery rates on *R*_*e*_, final herd prevalence, the average duration of the first introduction of infection to the herd (mean duration of infection), the case fatality proportion, the proportion of cases that resolve through the formation of sequestra, and the average number of cases, animals that form sequestra and animals that succumb to CBPP is presented in Table 7. A 50% reduction in *R*_*e*_ caused mortality to decline from 179 to 76 deaths, 57.5% decline. Note that reducing *R*_*e*_ to 1.42 was consistent with a final herd prevalence of 3.8%. In cases where *R*_*e*_ was near or below 1, the disease faded out entirely from all 500 iterations of the simulation.

The results of elective treatment applied in only one of the three herds are presented in Table 8. Reduction of the infectious period to 25% of the baseline value reduced the herd level prevalence moderately to 59.6%. On the other hand, mean mortality was reduced by more than 75% to 45 from 179 head.

**Figure 8:**
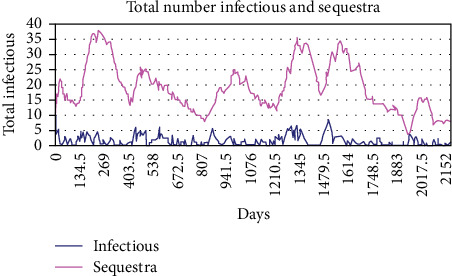
Total number of infectious and sequestra resulting when the duration of the infectious period is set to 3.5 days. When treatment was modeled, the parameter for the duration of the infectious period was reduced, but the duration of the sequestra was not changed. Although the total number of infectious individuals and individuals with sequestra was reduced, the ratio of sequestra to infectious individuals present in the population was increased.

Although the total number of animals that developed sequestra was reduced for the various values of the duration of infection, the ratio of animals in the sequestra state to those in the infectious state was increased in these treatment models (Figure 8).

#### 3.2.1. Integrated Programs of Treatment and Vaccination

The results of scenarios modeling the integration of treatment and vaccination across all three populations are presented in Tables 9–12. Note that for each vaccination scenario (row in the tables), the reduction of the infectious period greatly reduced the final herd prevalence and total mortality. For example, in Table 9, a program on annual vaccination for 2 years resulted in a very modest reduction of herd prevalence of 4.0% from 79.6% in the baseline to 75.6%. Integration of antibiotic treatment with the effect of reducing the infectious period to 25% of the baseline reduced prevalence by 76% from 79.6% in the baseline to 3.6%. Integrated treatment and vaccination programs where the vaccination is of moderate duration of immunity (2–3 years) interrupted transmission entirely (Tables 11 and 12).

The results of the simulation of an elective program of integrated treatment and vaccination are presented in Table 13. The effect of a rigorous program of treatment and annual vaccination (85%–95% efficacy and 3-year duration of immunity) reduced herd level prevalence to 49.8% and mortality to seven deaths in the reference herd over the 6-year life of the simulation. Treatment alone in elective programs (Table 8) reduced deaths to 20 over a 6-year period.

## 4. Discussion

The SVEIQR models were published over a decade ago, and the results provoked robust discussion. The validation of the models and results in terms of the dynamics of transmission in the absence of control interventions is comprehensively described in previous publications and will not be repeated here. More recently, Aligaz and Mungunga [20] published a detailed mathematical analysis of the models with minor enhancements on the SVEIQR approach and found similar results on disease dynamics and the impact of treatment.

The original SVEIQR models used standard exponential decays for the loss of vaccinal immunity, which underestimated the impact of vaccination. To compensate for this, generous assumptions (a most likely value of 3 years in the PERT distributions) were made regarding the duration of immunity in the original model papers. For the current work, the authors updated the models to use more appropriate gamma distributions as the shape of the loss of immunity. As illustrated in Figures 2–4, they give a more appropriate shape to the decay. In order to assess the impact of the changes, simulations of the impact of vaccination are given in Table 4 for some of the same scenarios (input parameter settings) as in the original research [4]. The results of the annual vaccination programs were largely similar, while the gamma decay predicted a much greater impact for the biannual programs. For example, a 2-year biannual program with a vaccine with 50%–80% efficacy and 3 years duration of immunity predicted a final herd prevalence of 28.4% and 75 deaths in the gamma decay model, whereas the same conditions in the original model with the exponential decay predicted 57.2% herd prevalence and 129 deaths in the herd of 500 head over 6 years. Although the impact of vaccination was increased in the new model, it is important to note that only biannual programs of four to 5 years duration with a vaccine with 85%–95% efficacy and 3-year duration of immunity approached elimination of CBPP from herds of 500 head.

Aligaz and Munganga calculated from their model that vaccines could control the disease using a 3-year duration of immunity as we did in our original model. However, more importantly, they appear to have interchanged vaccination (injection of vaccine) with successful immunization [20]. In a vaccine with limited efficacy, this difference in approach results in major differences in estimates of the impact of vaccination.

The GALVmed-sponsored vaccine trial found an 87% reduction in severe lesions 6 months postvaccination [10]. In comparison with the extensive body of literature on the efficacy of CBPP vaccines, this result is at the upper limit. The historical literature on the efficacy and duration of immunity of the T1/44 vaccine is chaotic. In seven separate studies, at three to 6 months postvaccination, the efficacy scores of the vaccine in terms of protection against macroscopic lesions (*E*_*p*_) ranged between 33% and 95% [11–14, 16, 17, 21]. At 12–15 months postvaccination, three papers reported *E*_*p*_ values between 66% and 75% [12, 13, 17]. One paper reported 80% protection (*E*_*p*_) 2 years postvaccination [15].

For the purposes of the analysis, the authors chose to focus on two levels of efficacy. A PERT distribution with a range of 85%–95% with a most likely value of 90% was used to model the GALVmed vaccine trial results in isolation. Additional sets of simulations were run using PERT distributions with a range of 60%–90% with a most likely value of 75%. These values were an increase of 10% over the estimates used in the original modeling papers and were considered to represent the body of information amended for the new data.

Comparing Tables 4 and 5 and the results of the sensitivity analysis (Figure 5), the duration of immunity had the greatest influence on the impact of vaccination in terms of eliminating infection from the population.

The data on treatment extends back to 1961 when Novarsenobensol was shown to result in significant to complete clinical improvement and lowered the case fatality proportion [22]. Later, Hudson and Etheridge [23] showed that tylosin resulted in reductions in clinical disease and clearance of bacteremia. In 1971, Camara [24] reported that bronchocilline, aureomycine, and sanclomycine resulted in high rates of cure and suggested treatment of ill animals could complement vaccination. More recent studies on oxytetracycline have shown high recovery rates and reduced rates or an absence of sequestration in recovery animals [25–27].

The effect of interest in epidemiological analysis designed to assess control is the impact of treatment on transmission resulting in new *infectious* individuals. Information on the clinical course, shedding of the infection agent, pathology, and serology can be used as surrogates for data on transmission or as additional information to support the interpretation of data on transmission. Information from several studies on the impact of treatment on the clinical and pathological course of disease are available. Studies that directly access transmission from treated infected cattle to in-contact sentinels, such as the studies by Hubschle et al. [28] and Muuka et al. [2], are the most valuable.

There were options in the manner of modeling antibiotic treatment. The probability that a contact is infectious *p* is determined by the biological properties of the host and agent. One of the determinants of *p* is the rate an infectious agent is shed by an infected host. Antibiotic treatment affects the biological behavior of the agent, such as the amount of agent in the host, its distribution in host tissues, and the level or rate at which it is shed. Thus, effective antibiotic treatment may act by lower *p* without entirely clearing infection from the host or resulting in a sterile “cure.”

On the other hand, antibiotic treatment often does result in a cure. This can be modeled as a reduction in the duration of infection (*d* or 1/*α*).

Whether *p* or *d* is reduced, the effect is to reduce the effective reproductive rate *R*_*e*_ of the agent. If *R*_*e*_ is reduced to below 1, the agent will fade out and be eliminated from the population.

The effect of antibiotic treatment could be modeled by adjusting *p* and *d* or using data on shedding of the agent or by extrapolation from clinical data on symptoms and cure to determine the impact of treatment on transmission (*R*_*e*_) in various control packages. Preferably, data on transmission (numbers of secondary cases, etc.) can be used to directly calculate *R*_*e*_ and determine the impact on transmission.

In addition to the current GALVmed study on treatment with tulathromycin, gamithromycin, and oxytetracycline, the impact of treatment with danofloxacin [9] and oxytetracycline [27] on transmission has been studied. Danofloxacin resulted in a statistically significant reduction in transmission to in-contact controls. Niang did not find that CBPP was transmitted to contact animals from oxytetracycline-treated CBPP cases, which is also consistent with an *R*_*e*_ of less than 1. Thus, Muuka et al. [2] were not alone in finding third-generation macrolides and oxytetratcycline suppress CBPP transmission.

The GALVmed-sponsored antibiotic trials found no evidence of effective transmission to in-contact sentinels that resulted in new infectious hosts. There was evidence of transient infections where single indicators suggested some level of limited infection at some point in the experiment. Upon postmortem, two of the gamithromycin in-contact sentinels had resolved lesions without other evidence of infection. In the case of tulathromycin, one seroconversion without evidence of pathology was observed [2]. This finding was supported by detailed data showing a significant reduction in the pathology, clinical symptoms, and infection in the treated animals. This suggests a powerful reduction of transmission consistent with estimates of *R*_*e*_ of 1 or less. This level of efficacy is equivalent to the impact on transmission of removal of clinical cases by culling. Oxytetracycline was also shown to have beneficial effects consistent with reduction of *R*_*e*_ of less than 1. The experimentation in the model utilized *R*_*e*_ parameter values that resulted in a range of about four to less than one. This reflected the full range of potential antibiotic effects.

There are two principal approaches to applying antibiotics: mass treatment of all animals regardless of clinical status or selective treatment of identified cases. If well applied, the mass treatment approach should result in the treatment of all or nearly all infected animals. In fact, one field application of mass antibiotic treatment reported successful eradication of CBPP after a single intervention [29].

The modeling approach adopted in this report is useful to investigate either mass treatment or targeted treatment of clinical cases. The principal epidemiological difference between mass treatment and treatment of clinical cases is that mass treatment would also treat inapparent latent and sequestra cases. The latent period for CBPP is prolonged and it would be expected that clinically inapparent infection is present in most outbreaks of CBPP. The impact of treatment on animals incubating CBPP was not assessed. An important difference may exist between mass treatment and treatment targeted to clinically ill animals in this regard that was not captured in the current model experimentation.

Based on the review of the literature conducted in the original modeling studies [3], the available evidence suggests that sequestra rarely, if ever, return to an infectious state and are probably not of epidemiological significance. Despite the assumptions in textbooks and opinion literature that sequestra are a source of transmission, there is no documented evidence of the natural reactivation of sequestra and shedding of mycoplasmas in the observational literature and experimental attempts to reactivate sequestra have failed [30]. It is also important to note that although we modeled the development of sequestra as an outcome of recovery, rational treatment with antibiotics reduces the total number of sequestra in the population as it reduces the prevalence of cases. Thus, the population effect of treatment is to reduce the risk of sequestra.

Global Action Plan on Antimicrobial Resistance seeks to reduce overall antibiotic use but in no way suggests that clinically ill individuals or animals should be denied treatment where effective options exist [31]. Mass treatment with antibiotics was not specifically investigated in the modeling, considering the international initiatives to limit unnecessary antibiotic use.

The efficacy of clinical treatment is dependent in part on the accurate identification of cases. In most areas where CBPP is endemic, livestock owners and animal health service delivery actors are astute in the clinical diagnosis of CBPP [32, 33]. The most common diagnostic problem is confusion of CBPP with other pneumonias. This should not be considered a problem in practical terms or in relation to policies to limit antibiotic use: treatment with third-generation macrolides is appropriate for any bacterial pneumonia and common practice in the production systems of developed countries.

The results reported for the two third-generation macrolides and oxytetracycline suggest that prompt treatment of all clinical cases in a herd could essentially eliminate CBPP from a herd. A single treatment pass would be of major benefit but would probably not eliminate the infection. Given that animals in the early stages of inapparent infection are probably present, the herd would need to be closely monitored for up to 6 months, and additional cases treated as they appeared to achieve elimination.

Oxytetracycline is widely available and is widely used by both veterinarians and livestock owners in the developing world in the treatment of bovine pneumonias, including CBPP. The treatments are usually delivered in an opportunistic manner to severe cases and are not part of a systematic clinical treatment or control effort. Both veterinarians and livestock owners report that they use oxytetracycline in the treatment of CBPP because it gives positive results. One concern is that suboptimal treatment strategies will result in resistance to the oxytetracycline; however, the published oxytetracycline trials [1, 2, 34] against CBPP indicate that the drug continues to be highly efficacious in the treatment of CBPP.

The results in Table 7 indicate that the average infectious period was reduced to 10.5 days by a coordinated program of treatment. This would require clinical cases to be recognized and treated promptly. It is possible to envisage a program implemented by specifically trained community animal health workers who visited every herd in an area once a week and treated all suspicious pneumonias. It is reasonable to hypothesize that such a program could eliminate CBPP from a community within 6 months. Livestock owners are willing to implement CBPP control when good information and effective tools are made available [35, 36]. This approach is attractive as it suggests that targeted programs that are focused spatially and temporally, as were used for rinderpest eradication, could be possible for CBPP.

The results presented in Tables 9–12 suggest integrated programs of vaccination and treatment are a feasible option that will lead to elimination over a period of years. Reductions of 50%–75% in the duration of the infectious period are attainable with less rigorous programs of treatment. Sustaining mass vaccination with 80% coverage and 80% efficiency over 3 years under current socioeconomic and institutional conditions has proved elusive but should be possible if a reasonably funded public program was initiated.

The experiments where interventions were applied to only one of the three herds to mimic elective control programs where one livestock keeper chooses to invest in CBPP control, but his neighbors do not demonstrate important reductions in mortality (and morbidity) that are of economic benefit to the private individual. Regular vaccination or treatment or both also resulted in fewer new cases of CBPP in the herd. Perhaps the best evidence of the utility of this approach is the widespread, daily decision taken by livestock owners and veterinarians to treat CBPP with oxytetracycline.

Rigorous biannual vaccination reduced the mortality experienced from 179 deaths in the baseline model to 41 deaths over a 5 year period of vaccination. An active and prompt treatment program could achieve a reduction to as low as 20 deaths. A combination of these options only resulted in the elimination of a further 13 deaths to a level of seven deaths over the 6-year period. Thus, in terms of outcomes, all three programs were similar, and the additional reduction of 13 deaths in the combined program may not be economically justified. A simple benefit–cost analysis should indicate which is the best choice among elective treatment, vaccination, or combined treatment and vaccination.

The results of the modeling suggest that selected intervention options should be piloted at community scale. The research findings on third-generation macrolides and oxytetracycline are game changers. A simple, effective treatment that results in dramatic clinical improvement (private benefit) and stops transmission in its tracks (public good) completely changes the nature of the challenge of CBPP. The clear and immediate clinical response to treatment can act as an important incentive for individuals to invest in CBPP control.

The modeling suggests that pilot programs of appropriate treatment, appropriate treatment combined with vaccination and vaccination alone should be undertaken and compared. A control group should be included where no specific intervention package is promoted, but normal animal health market activities are permitted. The vaccination alone group with currently available vaccines is proposed as a reference group. The ideal location would be an area with more or less stable endemic disease. Areas, where CBPP is sporadic or occurs in epidemics, would lead to results that are more difficult to analyze. The objective (control vs elimination) would need to be explicitly defined and appropriate design features and impact measurement would relate to the objective. In the case of control or suppression, as complement fixation is an indicator of active infection, trends in CFT CBPP prevalence could be an affordable measurement tool. More in-depth study of selected individuals from the populations, including postmortems, could be considered. Studies that set out to document elimination would require a longer duration of follow-up as well as monitoring of neighboring communities. The challenge would be to differentiate recurrence from reintroduction if cases should occur. A research organization or university experienced in fieldwork in Africa in partnership with private service providers, a non-governmental organization, and the national veterinary services would bring together the right combination of skills and experience to implement the study.

For treatment, it is proposed that community animal health workers operating under the supervision of veterinarians be specifically trained to recognize and treat CBPP cases according to a defined protocol. This model could be compared to other service delivery models. The pilots should be closely monitored in terms of the impact of control measures on the incidence of CBPP, cost, incentives for participation, and the emergence of antibiotic resistance. Antibiotics result in immediate and clear private benefits that can drive CBPP control and eradication through private investment, thus overcoming the lack of public sector investment in CBPP control that has occurred over the past several decades.

## 5. Conclusions

Treatment of mycoplasmal pneumonias in cattle with antibiotics is standard practice throughout most of the world. The results presented in this paper for the impact of treatment and vaccination of CBPP are consistent with previous reports in the literature. Vaccination with current vaccines is unlikely to have a significant impact on the prevalence of CBPP unless combined with other interventions such as treatment. Systematic antibiotic treatment of infected animals or combined antibiotic treatment and vaccination programs are capable of controlling and eliminating infection. Oxytetracycline and third-generation macrolides were equally effective in interrupting transmission. The macrolides were more effective in controlling clinical signs, which could act as an incentive to promote treatment. On the other hand, oxytetracyclines are considerably cheaper and already widely available. The development and validation of practical treatment or treatment and vaccination protocols have the potential to reduce overall antibiotic use and lead to more effective antibiotic stewardship.

## Figures and Tables

**Table 1:** Parameter definitions and default values for a spatially heterogeneous SVEIQR model of contagious bovine pleuropneumonia transmission in pastoral communities of East Africa [4].

Parameter	Definition	Min	Mode	Max
A	Amplitude of seasonal forcing	—	0.5	—
*α_q_*	Rate of sequestrum formation	0.011	0.013	0.018
*α*_*r*_	Rate of recovery	0.0036	0.0045	0.0059
*β*_win_	Effective within-group contact rate	0.07	0.126	0.127
*β*_bet_	Effective between-group contact rate	—	*ηβ*_win_	—
*E*	Initial prevalence of exposed	0.01	0.02	0.03
*ε*	Vaccination efficiency	—	0.8	—
*η*	Ratio of between to within-group contact	—	0.1	—
*γ*	Transition rate from exposed to infectious	0.018	0.024	0.036
*I*	Initial prevalence of infectious	0.01	0.02	0.03
*κ*	Rate of sequestrum reactivation	0.00007	0.00009	0.00011
*μ*	Nonspecific mortality rate	0.00049	0.00055	0.00062
*N*	Subpopulation size	—	500	—
*p*_*e*_	Vaccine efficacy	0.5	0.65	0.8
*p*_*v*_	Proportion vaccinated	—	0.8	—
*R*	Initial prevalence of recovered	0.50	0.55	0.60
*ρ*	Proportion immunized	—	—	—
*σ*	CBPP-specific mortality rate	0.0064	0.009	0.013
*ω*_*n*_	Rate of loss of natural immunity	—	0.00027	—
*ω_v_*	Rate of loss of vaccinal immunity	—	Gamma decay (Figures 2–4)	—
*ψ*	Rate of sequestrum resolution	0.0068	0.0075	0.0079

*Note:* All rates are in units of days^−1^. The effective within-group contact rate (*β*) was equal to the physical contact rate (*C*) multiplied by the probability of transmission per contact (*p*) within subpopulations. The proportion immunized (*ρ*) was the proportion of animals vaccinated effectively out of all animals vaccinated. Loss of vaccinal immunity (*ω_v_*) was model as a series of eight serial decay states.

**Table 2:** Summary of indicators of infection as a proportion of the sentinel cattle placed in contact with treated cattle infected with the Afade strain of CBPP in the GALVmed study in Kenya [4].

Afade strain contact group	*N*	Fever ≥39.5°C	PM lesions	Culture from lung tissue	Serology (CFT)	*Effective* transmission
Saline controls	5	0.2	0.2	0.2	0.2	0.2
Tulathromycin	5	0	0	0	0.2	0
Gamithromycin	5	0	0	0	0.2	0
Oxytetracycline	5	0	0	0	0	0

*Note:* The table reflects the outcomes in sentinel animals placed in contact with each of the four treatment groups that had been previously placed in-contact with cattle infected by intubation. The saline control group was exposed, untreated cattle. The tulathromycin, gamithromycin, and oxytracycycline were exposed and treated with the designated antibiotic. *N* equaled the number of sentinels exposed to each treatment group 30 days after treatment. Transmission was the proportion of sentinel group showing evidence of *effective* transmission from the treatment groups based on four criteria: fever, lesions, culture of *M. mycoides*, and serology. A maximum complement fixation titer (CFT) of 10 was observed in one animal treated with gamithromycin and one treated with tulathromycin.

**Table 3:** Summary of indicators of infection as a proportion of the sentinel cattle placed in contact with treated cattle infected with the Caprivi strain of CBPP in the GALVmed study in Zambia [4].

Caprivi strain contact group	*N*	Fever ≥39.5°C	PM lesions	Culture from lung tissue	Serology (CFT)	*Effective* transmission
Saline controls	5	0	0.4	0.4	0.4	0.4
Tulathromycin	4	0	0	0	0.25	0
Gamithromycin	4	0	0	0	0	0
Oxytetracycline	5	0	0	0	0	0

*Note:* The table reflects the outcomes in sentinel animals placed in contact with each of the four treatment groups that had previously been placed in-contact with cattle infected by intubation. The saline control group was exposed, untreated cattle. The tulathromycin, gamithromycin, and oxytracycycline were exposed and treated with the designated antibiotic. *N* equaled the number of sentinels exposed to each treatment group 30 days after treatment. Transmission was the proportion of sentinel group showing evidence of *effective* transmission from the treatment groups based on four criteria: fever, lesions, culture of *M. mycoides*, and serology. *M. mycoides* was isolated from nasal swabs from one sentinel animal exposed to the oxytetracycline-treated group.

**Table 4:** Vaccination program results assuming a 1-year duration of immunity (*ω_v_* set to 0.02).

Frequency and duration of vaccination	Vaccine efficacy	Herd prevalence (%)	Mean duration of infection (days)	Mean total mortality (head)
No vaccination	0.5–0.8	77.6	1962	179
Annual—5 years		—	—	—
Biannual—2 years		—	—	—
Biannual—3 years		—	—	—
Biannual—4 years		—	—	—
Biannual—5 years		—	—	—

Annual—5 years	0.6–0.8	—	—	—
Biannual—5 years		—	—	—

Annual—2 years	0.6–0.9	85.8	2065	176.9
Biannual—2 years		77.6	2002	161.9

Biannual—5 years	0.7–0.8	—	—	—

Annual—2 years	0.85–0.95	83.0	2059	171.8
Annual—3 years		82.0	2048	164.7
Annual—4 years		87.0	2031	160.4
Annual—5 years		80.4	1994	154.5
Biannual—2 years		71.0	1918	148.8
Biannual—3 years		68.2	1842	131.2
Biannual—4 years		69.2	1812	125.9
Biannual—5 years		61.8	1786	114.4

*Note:* The table provides the final percentage of infected herds, the mean duration of infection in days, and the mean mortality for different vaccination programs with assumed ranges of vaccine efficacy as indicated. A dash in the output cells of the table indicates that the simulation for that combination of vaccination program was not run.

**Table 5:** Vaccination program results assuming a 3-year duration of immunity (*ω_v_* set to 0.005).

Frequency and duration of vaccination	Vaccine efficacy	Herd prevalence (%)	Mean duration of infection (days)	Mean total mortality (head)
No vaccination	0.5–0.8	77.6	1962	179
Annual—5 years		61.0	1725	126.1
Biannual—2 years		28.4	1218	75.0
Biannual—3 years		17.4	1086	59.6
Biannual—4 years		9.4	1016	51.1
Biannual—5 years		5.6	1000	46.0

Annual—5 years	0.6–0.8	39.0	1483	78.3
Biannual—5 years		4.0	937	41.6

Annual—2 years	0.6–0.9	59.4	1774	117.3
Biannual—2 years		18.4	1041	56.5

Biannual—5 years	0.7–0.8	2.2	874	37.1

Annual—2 years	0.85–0.95	43.8	1434	89.6
Annual—3 years		32.4	1252	66.5
Annual—4 years		23.2	1168	55.2
Annual—5 years		17.0	1143	45.5
Biannual—2 years		12.6	877	40.3
Biannual—3 years		2.2	749	28.7
Biannual—4 years		1.0	734	27.2
Biannual—5 years		0.4	734	26.6

*Note:* The table provides the final percentage of infected herds, the mean duration of infection in days, and the mean mortality for different vaccination programs covering all three herds with assumed ranges of vaccine efficacy as indicated.

**Table 6:** The impact of elective vaccination of the reference herd only on disease persistence and mortality in the spatially heterogeneous SVEIQR model of contagious bovine pleuropneumonia transmission in pastoral communities of East Africa.

Vaccination program	Herd prevalence (%)	Mean duration of infection	Mean total mortality
No vaccination	77.6	1962	179
Annual—5 years	60.6	1743	66.5
Biannual—2 years	68.2	1716	94.9
Biannual—3 years	62.4	1644	82.1
Biannual—4 years	61.6	1648	65.2
Biannual—5 years	53.0	1569	41.0

*Note:* The table provides the final herd prevalence, the mean duration of infection in days, and the mean total mortality for different vaccination programs. The proportional vaccine efficacy was 0.85–0.95, the proportion vaccinated was 0.9, the vaccination efficiency was 0.9, and the duration of immunity was 3 years. The vaccination program column gives the frequency and duration of campaigns. The herd prevalence column gives the percent of reference herds that had at least one infected (infectious or sequestra) animal at the end of the 6-year simulation. Note that in calculating the mean duration of infection, in the reference herds where the disease did not fade-out, the duration was truncated at 2141 days (~6 years).

**Table 7:** The results of simulations of systematic treatment of clinical cases across the population.

Duration (1/*α*)	*R*_*e*_	Herd prevalence (%)	Mean duration of infection	Case fatality proportion	Case sequestration proportion	Mean total cases	Mean total sequestra	Mean total mortality
56	4.10	79.6	1968	0.33	0.49	541	268	179
42	3.34	62.4	1824	0.27	0.54	476	260	130
28	2.56	42.2	1590	0.21	0.58	360	216	76
21	2.01	22.4	1257	0.17	0.59	228	147	39
14	1.42	3.8	811	0.13	0.58	87	64	11
10.5	1.07	0.0	600	0.11	0.57	42	35	4
7	0.76	0.0	530	0.09	0.55	26	25	2

*Note:* The effect of reducing the duration of the infectious period in all herds from 56 to 7 days on the persistence of infection in the spatially heterogeneous SVEIQR model of contagious bovine pleuropneumonia transmission in pastoral communities of East Africa. The effect of reducing the duration of the infectious period, one of the effects of treatment, on the effective *R* (*R*_*e*_), final herd prevalence, mean duration of infection of herds, case fatality proportion, the proportion of case sequestration that resolves as sequestra, mean total cases, mean total sequestra and mean total mortality is shown. Note that even though the case sequestration rate increases, treatment at the population level reduces the number of sequestra that occur in the population due to the reduction in the number of cases. The values of *R*_*e*_ presented consider only transmission within the reference population. The herd prevalence column gives the percent of reference herds that had at least one infected (infectious or sequestra) animal at the end of the 6-year simulation. The case sequestration proportion is the proportion of infected cases that are resolved through the process of sequestra formation. The mean total number of sequestra is the total number of sequestra formed in the reference herd over the 6-year duration of the simulation. The more efficacious treatment scenarios resulted in eradication.

**Table 8:** The results of simulations of elective treatment in one herd while in-contact herds do not take action.

Duration (1/*α*)	*R*_*e*_	Herd prevalence (%)	Mean duration of infection	Case fatality proportion	Case sequestration proportion	Mean total cases	Mean total sequestra	Mean total mortality
56	4.10	79.6	1968	0.33	0.49	541	268	179
42	3.34	75.0	1919	0.27	0.54	516	281	141
28	2.53	69.8	1886	0.21	0.59	477	286	99
21	1.95	62.2	1828	0.16	0.62	428	274	69
14	1.42	59.6	1777	0.12	0.65	378	255	45
10.5	1.08	57.2	1792	0.11	0.57	343	240	31
7	0.76	57.8	1771	0.06	0.69	313	227	20

*Note:* The effect of reducing the duration of the infectious period from 56 to 7 days in the reference herd only on the persistence of infection in the spatially heterogeneous SVEIQR model of contagious bovine pleuropneumonia transmission in pastoral communities of East Africa. The effect of reducing the duration of the infectious period in the reference herd only, one of the effects of treatment, on the effective *R* (*R*_*e*_), final herd prevalence, mean duration of infection of herds, case fatality proportion, the proportion of case sequestration that resolve as sequestra, mean total cases, mean total sequestra and mean total mortality is shown. Note that even though the case sequestration proportion increases, treatment at the population level reduces the number of sequestra that occur in the population due to the reduction in the number of cases. The values of *R*_*e*_ presented consider only transmission within the reference population. The herd prevalence column gives the percent of reference herds that had at least one infected (infectious or sequestra) animal at the end of the 6-year simulation. The mean total number of sequestra is the total number of sequestra formed in the reference herd over the 6-year duration of the simulation. The main benefit is a large reduction in the mortality due to CBPP in the treated herd from CBPP.

**Table 9:** The effect of simultaneous vaccination with a vaccine of efficacy 60%–90% and 2 years duration of immunity and reductions in the infectious period (1/*α*) as an effect of treatment on persistence of infection and mortality in the spatially heterogeneous SVEIQR model of contagious bovine pleuropneumonia transmission in pastoral communities of East Africa.

Vaccination program	Baseline 1/*α*		50% 1/*α*		25% 1/*α*	
	Prevalence	Mortality	Prevalence	Mortality	Prevalence	Mortality
None	79.6	179	42.2	76	22.4	39
Annual—2 years	75.6	159	19.6	34	3.6	11
Annual—3 years	—	—	—	—	2.0	10
Annual—5 years	—	—	—	—	0.2	9
Biannual—2 years	49.2	111	5.0	18	1.4	9
Biannual—3 years	43.4	92	—	—	0.4	8
Biannual—5 years	28.6	66	0.4	14	0.0	8

*Note:* The proportion vaccinated was 0.8, and vaccination efficiency was 0.8. Final herd prevalence was defined as the percent of reference herds that had at least one infected (infectious or sequestra) animal at the end of the 6-year simulation. A dash in the output cells of the table indicates that the simulation for that combination of vaccination program and treatment impact was not run. Note that treatment of infectious cases greatly augmented impact and combined programs could eradicate the disease.

**Table 10:** The effect of simultaneous vaccination with a vaccine of efficacy 60%–90% and 3 years duration of immunity and reductions in the infectious period (1/*α*) as a result of treatment on persistence of infection and mortality in the spatially heterogeneous SVEIQR model of contagious bovine pleuropneumonia transmission in pastoral communities of East Africa.

Vaccination program	Baseline 1/*α*		50% 1/*α*		25% 1/*α*	
	Prevalence	Mortality	Prevalence	Mortality	Prevalence	Mortality
None	79.6	179	42.2	76	22.4	39
Annual—2 years	59.4	117	7.2	20	1.4	9
Annual—3 years	—	—	3.4	17	1.0	8
Annual—5 years	—	—	1.0	15	0	8
Biannual—2 years	18.4	57	0.8	14	0	7
Biannual—3 years	—	—	0	13	—	—
Biannual—5 years	1.8	37	0	13	—	—

*Note:* The proportion vaccinated was 0.8, and vaccination efficiency was 0.8. A dash in the output cells of the table indicates that the simulation for that combination of vaccination program and treatment impact was not run. Prevalence was defined as the percent of reference herds that had at least one infected (infectious or sequestra) animal at the end of the 6-year simulation. Note that treatment of infectious cases greatly augmented impact, and combined programs could eradicate the disease within 2 years.

**Table 11:** The effect of simultaneous vaccination with a vaccine of efficacy 85%–95% and 2 years duration of immunity and reductions in the infectious period (1/*α*) as a result of treatment on persistence of infection and mortality in the spatially heterogeneous SVEIQR model of contagious bovine pleuropneumonia transmission in pastoral communities of East Africa.

Vaccination program	Baseline 1/*α*		50% 1/*α*		25% 1/*α*	
	Prevalence	Mortality	Prevalence	Mortality	Prevalence	Mortality
None	79.6	179	42.2	76	22.4	39
Annual—2 years	71.8	144	13.0	25	2.2	9
Annual—3 years	70.4	128	5.6	17	0.6	8
Annual—5 years	61.4	106	3.0	14	0.2	8
Biannual—2 years	35.6	82	1.6	12	0.4	7
Biannual—3 years	25.6	60	1.0	11	0.2	7
Biannual—5 years	13.6	42	0.0	11	0.0	7

*Note:* The proportion vaccinated was 0.8, and vaccination efficiency was 0.8. Prevalence was defined as the percent of reference herds that had at least one infected (infectious or sequestra) animal at the end of the 6-year simulation. Note that treatment of infectious cases greatly augmented impact, and combined programs approached eradication of the disease within 2 years.

**Table 12:** The effect of simultaneous vaccination with a vaccine of efficacy 85%–95% and 3 years duration of immunity and reductions in the infectious period (1/*α*) as a result of treatment on persistence of infection and mortality in the spatially heterogeneous SVEIQR model of contagious bovine pleuropneumonia transmission in pastoral communities of East Africa.

Vaccination program	Baseline 1/*α*		50% 1/*α*		25% 1/*α*	
	Prevalence	Mortality	Prevalence	Mortality	Prevalence	Mortality
None	79.6	179	42.2	76	22.4	39
Annual—2 years	43.8	90	2.8	14	0.4	7
Annual—3 years	32.4	67	—	—	—	—
Annual—5 years	17.0	46	0.2	12	0.0	7
Biannual—2 years	12.6	40	0.6	11	0.0	7
Biannual—3 years	2.2	29	0.0	10	—	—
Biannual—5 years	0.4	27	0.0	11	—	—

*Note:* The proportion vaccinated was 0.8, and vaccination efficiency was 0.8. A dash in the output cells of the table indicates that the simulation for that combination of vaccination program and treatment impact was not run. Prevalence was defined as the percent of reference herds that had at least one infected (infectious or sequestra) animal at the end of the 6-year simulation. Note that treatment of infectious cases greatly augmented impact, and combined programs could eradicate the disease within 2 years.

**Table 13:** The effect of reducing the duration of the infectious period from 56 to 1.75 days and simultaneous annual vaccination with a vaccine of 85%–95% efficacity and a 3-year duration of immunity *in the reference herd only* on the persistence of infection in the spatially heterogeneous SVEIQR model of contagious bovine pleuropneumonia transmission in pastoral communities of East Africa.

Duration (1/*α*)	*R*_*e*_	Program	Herd prevalence (%)	Mean duration of infection	Case fatality proportion	Case sequestration proportion	Mean total cases	Mean total sequestra	Mean total mortality
42	3.33	2 years annual	67.4	1826	0.28	0.53	351	193	96
42	3.33	5 years annual	55.4	1682	0.29	0.52	193	107	54
28	2.54	5 years annual	55.0	1695	0.22	0.57	168	104	36
14	1.95	5 years annual	52.6	1655	0.17	0.60	147	97	25
7	1.42	5 years annual	49.0	1624	0.13	0.63	128	91	16
3.5	1.06	5 years annual	50.2	1650	0.10	0.64	120	88	11
1.75	0.80	5 years annual	49.8	1616	0.07	0.66	111	85	7

*Note:* The results of results reflect elective treatment in one herd while in-contact herds do not take action. The proportion vaccinated was 0.8 and vaccination efficiency was 0.8. A dash in the output cells of the table indicates that the simulation for that combination of vaccination program and treatment impact was not run. Prevalence was defined as the percent of reference herds that had at least one infected (infectious or sequestra) animal at the end of the 6-year simulation. Note that treatment of infectious cases augmented impact and combined programs reduced the number of sequestra cases and the herd mortality experience.

## Data Availability

Queries relating to the modeling parameters used in the publication can be addressed to the lead author (J Mariner). The information used in the models has been previously published; see appropriate references in this paper and available from the GALVmed document repository https://galvdox.galvmed.org/.
